# From Stress Pathways to Space Biology: An Odyssey in Molecular and Translational Medicine

**DOI:** 10.3390/biomedicines13102572

**Published:** 2025-10-21

**Authors:** Alkmini T. Anastasiadi, Vassilis L. Tzounakas

**Affiliations:** Department of Biochemistry, School of Medicine, University of Patras, 26504 Patras, Greece; aanastasiadi@upatras.gr

Nowadays, molecular and translational medicine can be seen as a journey across vast and sometimes challenging landscapes of discovery. Each step forward brings new insights but also new questions and unexpected directions. In recent years, there is growing interest towards elucidating the mechanistic basis of pathophysiological backgrounds [1], identifying accurate and easy-to-detect biomarkers [2], and designing novel therapies [3,4] that are not only promising but also adaptable to individual patients. Hence, the convergence of high-throughput technologies, sophisticated computational approaches, and integrative biological models has opened the doors to a new scientific era of unprecedented acceleration. The goal of this Special Issue was to capture the momentum and showcase European contributions that reflect both deep mechanistic exploration and broad translational application. The seven articles gathered here illustrate how diverse journeys can meet at a common destination: advancing human health.

One such voyage shows how mechanistic dissection at the cellular level can reveal therapeutic perspectives. Bourouti et al. [5] investigate the impact of hyperosmotic stress on cardiac myoblasts and show that phosphorylation of eIF2α depends on oxidative stress and Na^+^/H^+^ exchanger-1 activity, while being independent of MAPK activation. By distinguishing the protective roles of autophagy and p38-MAPK signaling from the pro-apoptotic drive of ER stress, their work sheds light on independent yet converging pathways of survival and death, pointing toward new possible targets for cardiovascular intervention. From the cellular perspective, the journey continues into the shifting landscapes of cancer. Salomon-Perzyński et al. [6] apply targeted next-generation sequencing to multiple myeloma, including patients with advanced and relapsed disease. Their comprehensive genomic profiling identifies dynamic clonal evolution and druggable mutations while revealing distinct patterns with prognostic significance. Perhaps most importantly, they show that reassessing the Revised International Staging System (R-ISS) at relapse refines patient stratification, reminding us that continuous genomic monitoring can sharpen therapeutic decisions in real time.

Yet not all destinations are confined to Earthly shores. Space biomedicine is an emerging field that investigates how unique extreme conditions, such as microgravity, cosmic radiation, isolation, and confined environments, affect human physiology [7,8]. Research in this area has already provided valuable insights into muscle and bone loss, cardiovascular adaptation, immune function, and cellular biology, often leading to innovations that return to Earth as diagnostic or therapeutic strategies. Bizzarri et al. [9] take this further by arguing that research in microgravity illustrates life as a non-equilibrium thermodynamic system. Here, weak forces, such as gravity, act as symmetry-breaking constraints that shape biological organization. This theoretical reappraisal pushes us beyond a strictly gene-centered model, offering space biomedicine as a fertile ground for new conceptual frameworks and biomedical applications.

Other contributions provide us with tools that refine clinical navigation. In precision medicine, progress is not always about inventing something completely new; it often comes from applying or combining established methods in novel ways. In other words, in biomedicine you can “teach an old dog new tricks”, harnessing familiar tools with fresh perspectives to extract clinically meaningful insights. In a context like this, Turchin et al. [10] introduce a multimodal optical platform that brings together diffuse reflectance spectroscopy, laser Doppler flowmetry, and optical coherence tomography/angiography to monitor burn wound grafts. Their findings show that blood oxygenation and perfusion can predict graft outcomes early, equipping clinicians with a non-invasive and reliable compass for personalized burn care. Similarly, Zaia et al. [11] describe the LOTO study, validating a fractal lacunarity-based MRI parameter (TBLβ) as a marker of trabecular bone quality. Unlike traditional bone mineral density, this measure captures microarchitectural fragility and responds to bisphosphonate therapy, providing a sensitive, non-ionizing, and clinically transferable method for monitoring osteoporosis.

Scientific advancement also comes through reimagining what already exists. Sometimes, as Odysseus demonstrated in the cave of Polyphemus, the answer lies in using familiar resources in unexpected and unknown-to-date ways [12,13]. A case in point is presented in the study by Matiș et al. [14], who explore the use of probiotics as psychobiotics in children with gastrointestinal comorbidities. They report improvements in hyperactivity, aggression, and concentration, while mapping how neurotransmitter and hormonal imbalances align with specific neuropsychiatric symptoms. Their findings highlight the potential of gut–brain axis modulation as a therapeutic strategy and emphasize the importance of non-pharmacological interventions in pediatric care. Finally, Hara et al. [15] review the use of urinary volatile organic compounds for non-invasive cancer detection. Surveying evidence from mass spectrometry, animal-based olfactory systems, and biosensors, they demonstrate how diverse methods converge toward rapid, accurate, and accessible cancer diagnostics. From nematode-based chemotaxis to graphene “electronic noses”, their synthesis underlines how molecular insights are steadily being translated into practical, patient-centered solutions.

Taken together, the contributions of this Special Issue form an odyssey of translational medicine extended through time (examination of time-dependent biological changes) and space (by extending research beyond the barriers of earthly habitat). Each study represents a waypoint on a longer journey: some illuminate molecular routes, others chart evolutionary avenues, while still others offer new navigational tools or open entirely new horizons. Just as Cavafy reminds us that the value of Ithaca lies in the journey itself [16], the studies presented here show that the progress of translational medicine is as important as its endpoints. Each discovery, each tool, and each perspective enriches the path, making the scientific odyssey itself the true reward.
